# Pupillary response to moving stimuli of different speeds

**DOI:** 10.16910/jemr.14.1.3

**Published:** 2021-12-23

**Authors:** Yuexin Wang, Yining Guo, Jiajia Wang, Ziyuan Liu, Xuemin Li

**Affiliations:** Peking University Third Hospital, Beijing, China; These authors contributed equally to the article.; Corresponding author

**Keywords:** Motion perception, pupillary response, photoreceptor, eye movement, eye tracking, saccades, smooth pursuit

## Abstract

To investigate the pupillary response to moving stimuli of different speeds and the influence of different luminance environments, 28 participants with normal or corrected-to-normal vision were included. The participants were required to track moving optotypes horizontally, and their pupils were recorded on video with an infrared camera. Stimuli of different speeds from 10 to 60 degree per seconds were presented in low (0.01 cd/m2) and moderate (30 cd/m2) luminance environments. Experiment 1 demonstrated that the motion stimuli induced pupil dilation in a speed-dependent pattern. The pupil dilation increased as the speed increased, and the pupil dilation gradually increased, then reached saturation. Experiment 2 showed that a stimulus targeting the rod- or cone-mediated pathway could induce pupil dilation in a similar speed-dependent pattern. The absolute but not relative pupil dilation in the cone paradigm was significantly larger than that in the rod paradigm. As the speed increased, the pupil dilation in the cone paradigm reached saturation at speed slower than the rod paradigm. Motion stimuli induced pupil dilation in a speed-dependent pattern, and as the motion speed increased, the pupil dilation gradually increased and reached saturation. The speed required to reach saturation in the cone paradigm was slower than in the rod paradigm.

## Introduction

Visual motion perception defines the processes that subjects use to identify the movement of themselves and the objects in their surroundings (21; 45; 22) and through visual input pathways (42). Motion perception via the visual system provides us with an enormous amount of information about changes in the environment, which is crucial for daily life tasks, such as sports performance and driving (31; 30). The processing of visual motion perception begins in the retina where the visual signal is modulated into specified spatial-temporal light intensity signals and is subsequently reconstructed into a three-dimensional structure, step-by-step in the higher motion processing cortical areas, including the middle temporal area (MT), medial superior temporal area and so on (6).

The pupil size depends on a complex interaction between the sympathetic and parasympathetic pathways that result in the activation of the sphincter and dilator muscles. The parasympathetic pathway receives multiple inputs at the level of the Edinger-Westphal (EW) nucleus and pretectal olivary nucleus (PON) from the cortical and subcortical areas, and the sympathetic pathway, which originates from the hypothalamus, also receives projections from the cortex (7; 19; 38). Thus, the pupil response has the potential to reflect cortical activities in addition to regulate light flux (9). Previous research has indicated pupillary responses to sensory and nonsensory stimulations, including color perception, cognitive load, and music prception,. (5; 52; 28; 17; 34; 18; 29). A pupillary response was also demonstrated during the transition from incoherent to coherent motion (43). Together with direction, speed is one of the most important motion features, and electrophysiological research has demonstrated speed-dependent variations in the N2 amplitude of visual evoked potentials (25). However, the relationship between motion speed and pupillary responses to motion remains to be identified.

The retina mainly comprises three layers, cones and rods, bipolar cells and ganglion cells (35). Cones and rods are light-sensitive photoreceptors, and rods are specified for high sensitivity under dark conditions, while cone provides high acuity with color when light is abundant (1). Following transduction via bipolar cells, the signal is integrated into ganglion cells with spatial-temporal specificity. There are mainly three types of ganglion cells: parvocellular-projecting (P) cells, magnocellular-projecting (M) cells, and small-field bistratified cells (11). Previous research has demonstrated that P cells have a relatively slower axonal conduction velocity and finer perception of stationary objects (10). In contrast, M cells have a faster response rate and prefer to transmit high temporal frequency signals. Additionally, the percentage of active M cells from the total active M and P cells increases as the temporal frequency increases, which means that the M cell-mediated pathway might play a pivotal role in motion perception (46).

Studies have shown that rods primarily project into the M ganglion cell pathway, while their input to P pathway cells is weak (33). The research has indicated that rod-mediated signals might selectively contribute to motion perception processes since the MT area receives input mainly from the M ganglion cell-based pathway (23; 37). Considering that the functions of cones and rods are luminance specified, well-designed research was performed and demonstrated the variation in motion perception and velocity discrimination in different luminance environments (20; 48). Thus, the pupillary response to motion might differ in different luminance environments, but this remains to be clarified. In the present study, the pupil size was monitored while motion stimuli of different speeds were presented to the subjects to test the pupillary response to the motion stimuli and its speed dependence. Further research was performed under different luminance conditions to identify the difference in the pupillary response following motion signal input in the cone/rod-mediated paradigm.

## Methods

### Experiment 1

#### Participants

Twenty-eight healthy participants were enrolled for two experiements. Participants were eligible for inclusion in the study if they were 18 to 35 years of age with normal or corrected-to-normal vision. Observers with severe ametropia (diopter greater than +4D or -6D), retinopathy, glaucoma, cataract, corneal diseases, or cognitive disorders or if they could not see or track the motion targets without ametropia correction were excluded. The experiment was performed following the Declaration of Helsinki and approved by Peking University Third Hospital's ethical committee. All participants provided written informed consent before the study. Participants' personal information was well protected. The pupil data was not linked to a specific person.

#### Stimuli design

The stimuli were generated using MATLAB (2017b) and were displayed on a 12-inch LCD monitor with a frame rate of 60HZ with 2304x1440 resolution. The frame buffer depth was 24-bit (ARGB8888). The stimuli consisted of the fixation letter E in the middle of the screen and randomized moving letters (letter E/H/V/T/O). The font of the optotype letter E/H/V/T/O was from the Early Treatment of Diabetic Retinopathy Study [ETDRS] chart, and the size was 1 degree. The optotype was black and presented on a white background (main luminance 10 cd/m2).

**Figure 1.**
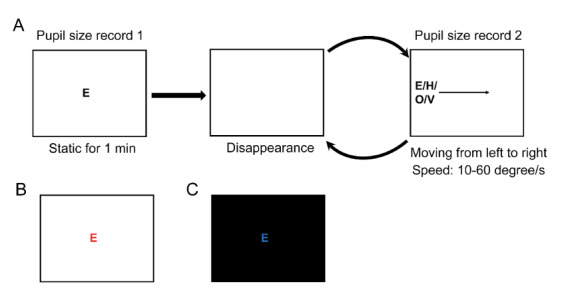
Paradigms and stimuli. The flow chart of the paradigm and stimuli in experiments 1 and 2. A. The flow chart of Experiment 1. B. The cone-mediated stimulus and background in experiment 2. C. The rod-mediated stimulus and background in experiment 2

The flow chart of the paradigm and stimuli were demonstrated in Figure 1. The experiment comprised six trials. Each trial began with the appearance of a fixation letter in the center of the screen. After 1 min, the fixation letter disappeared, immediately followed by the appearance of a moving letter. The moving letter moved horizontally from the center of the screen left border to the right border at a constant speed and disappeared, immediately followed by the appearance of the next moving letter. The appearance of the moving letter was repeated ten times at the same speed in each trial. The speed of the moving letter in six trials was 10 degrees/s, 20 degrees/s, 30 degrees/s, 40 degrees/s, 50 degrees/s or 60 degrees/s, respectively in a velocity-increasing sequence.

#### Procedure

Before the experiment, participants were informed of the instructions and then signed informed consent voluntarily. Afterward, dominant eye testing, automatic optometry, and uncorrected distance visual acuity (UDVA) (ETDRS, 5 m) measurements were conducted and recorded. The experiment was performed in a quiet room with a luminance of 10 cd/mm2. Participants were required to sit in front of the screen at 66 cm, and their head was fixed on a chin rest. The fixation of the head guaranteed the stability of the recording that avoided the target missing. We adjusted the sit to demonstrate the letter at the eye level. The non-dominant eye of each participant was monitored by an HD infrared camera (HYUNDAI, HY-K601) at a distance of 10 cm. The sampling rate of the camera was 30 Hz. The video resolution was 1080x720. The recording of the camera was started first, and a scale plate was temporarily placed at the outer canthus of the subject’s eyes before the presentation. The participant was instructed to stare at and pursue the static or moving letter as closely as possible during each trial. During the interval between each trial, subjects were allowed to blink or temporarily close their eyes for a rest, as they were required to keep their eyes fully open without blinking during each trial.

### Experiment 2

#### Stimuli design

The stimuli were generated and presented with the same software and monitor as Experiment 1. The stimuli consisted of a fixation letter E in the middle of the screen and a randomized moving letter (letters E/H/V/T/O). The letter font and size were the same as experiment 1, and the color of the letter and background was designed to target either the rod- or cone-mediated pathways shown in Figure 1B and Figure 1C. In brief, subjects were tested with the following two stimulus conditions: (1) For the rod stimuli, a short-wavelength blue light (dominant wavelength of 465 nm), low luminance optotype (0.01 cd/m^2^,) was presented on a black background (main luminance 0.01 cd/m^2^). (2) For the cone stimuli, a long-wavelength red light (dominant wavelength of 642 nm), moderate luminance optotype (30 cd/m^2^), was presented on a white background (main luminance 30 cd/m^2^). The experiment including the moving direction and speed of the stimuli targeting the rod- and cone-mediated pathways was the same as Experiment 1. The luminance and stimulus wavelength were confirmed at the location of participants’ eyes by a spectroradiometer (SpectraScan 740, Chatsworth, CA). Thus, each participant observed ten moving letters for each of 6 speeds in the cone and rod experiment, which was 120 moving letters.

#### Procedure

Participants were the same population as in experiment 1. The experiment targeting the rod-mediated pathway was performed in a dark room with luminance lower than 0.01 cd/mm^2^. Participants were required to sit in front of the screen at a distance of 66 cm. Participants were dark-adapted for 20 min before stimulus presentation. The setting, pupil monitoring and requirements for the participants were the same as in experiment 1. Following the paradigm for the rod-mediated pathway, subjects were photopically adapted for 10 min before the cone paradigm test began in a bright room with luminance higher than 10 cd/mm^2^.

#### Statistical analysis

The videos of each participant were thoroughly reviewed. When each moving letter initially appeared, the image of the pupil was manually captured by screenshot and used as the motion pupil. Thus, ten images were obtained initially for each trial. As for the static pupil, ten images over the last 20 seconds of 1 min when observing the fixation letter were captured evenly. According to previous studies, the pupil may constrict after blinks, and the pupil size will practically return to the baseline level after 2 seconds (26). Thus, images captured during 2 seconds after blinks were discarded. Images were also excluded if the participant did not pursue the target during the letter moving judged by the visual angle changes or if the upper eyelid covered more than half of the pupil on the video. For each trial, considering the exclusion criteria, the first five eligible images from ten captured images for motion and static pupils were included for the subsequent analysis. The pupil diameter of included images was measured with ImageJ (National Institutes of Health, Bethesda) calibrated by the scale on the plate placed before the stimuli presentation. Specifically, a line parallel to the line connecting the inner and outer canthus passing the corneal reflection point was drawn and intersect the limbus. And the line segment was regarded as the diameter of the pupil to be quantified automatically.

According to the result of repeated measures ANOVA (not shown), no significant differences were shown among the pupil diameter of five eligible images in one trial, indicating no evident adaptability in the pupil response during repeated stimulus presentations. Thus, the testing order would not be a confounding factor for the pupil response. The average pupil diameter from five eligible images was calculated for each trial as the static or motion pupil size for each subject. In some cases, due to an insufficient pursue of the moving letter or blinks, we could not collect enough images (less than 5) for a trial, and we took the average of the available (at least 4 episodes) images for statistical analysis.

Statistical data analysis was conducted using SPSS (Version 23.0). For the data from each participant, normalization was performed for the mean pupil diameter at each speed by subtracting or dividing the mean static pupil size. The absolute pupil dilation was calculated by subtracting the static pupil diameter from the motion pupil diameter in each trial, and the relative pupil dilation was obtained by dividing the motion pupil size by the static diameter. Considering the subject dependency, a linear mixed model was applied to analyze pupil diameter changes accounting for covariates. For experiment 1, a model was set to analyze the effect of speed on pupil diameter changes. And another model was established for experiment 2 to compared pupil diameter changes under different paradigms and speeds. The participants were set as subjects, and the speed was selected as a repeated factor and random intercept at the subject level was included for all models. The repeated covariance type was compound symmetry. Gender, dominant eye, age, spherical equivalent, and UDVA were adjusted. Bonferroni correction was applied when performing multiple comparisons to compare the main effect of different speeds.

## Results

Twenty-eight participants were included in the study with an average age of 22.2±2.65 years old, and 50% were male. Their average spherical equivalent was -2.81±2.37 diopter, and UDVA was 0.81±0.47. The dominant eye of 78.6% of participants was the right eye.

### Experiment 1

**Figure 2.**
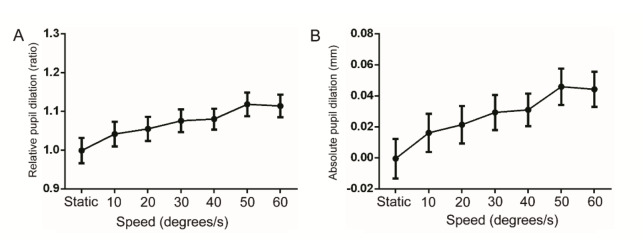
The relative pupil diameter with static optotypes and moving optotypes at the speeds of 10, 20, 30, 40, 50, and 60 degrees/s (n=28). A. The relative ratio of pupil diameters; B. Absolute value of the pupil diameter change.

The pupil diameter and pupil dilation across all speeds are shown in Table 1, and the p values of the comparisons between speeds on the relative and absolute pupil dilation are illustrated in Table 2. The motion stimulus induced significant pupil dilation, as demonstrated by the increasing absolute and relative pupil dilation at speeds of 10, 20, 30, 40, 50 and 60 degrees/s (p<0.05, respectively). The pupil dilation induced by the motion stimulus occurred in a speed-dependent manner, as the absolute and relative pupil dilation gradually increased as the speed increased, as shown in Figure 2. The post hoc analysis demonstrated that as the speed increased, the increase in the absolute and relative pupil dilation tended to reach saturation, as shown in Table 1 and the p-value in Table 2.

**Table 1.** Pupil diameter and pupil dilation of all speeds.

Speed	PD (mm)	APD (mm)	RPD (ratio)
(degree/s)	mean (SD)	mean (SD)	mean (SD)
Static	3.88 (0.65)	-0.005 (0.65)	1.0 (0.167)
10	4.04 (0.63)	0.16 (0.63)	1.04 (0.161)
20	4.09 (0.61)	0.21 (0.61)	1.05 (0.158)
30	4.17 (0.58)	0.29 (0.58)	1.08 (0.149)
40	4.19 (0.5)	0.31 (0.5)	1.08 (0.129)
50	4.34 (0.6)	0.46 (0.6)	1.12 (0.155)
60	4.32 (0.58)	0.44 (0.58)	1.11 (0.149)

Note: SD, standard deviation; PD, pupil diameter; APD, absolute pupil dilation; RPD, relative pupil dilation

**Table 2.** P-value of comparison between speeds for relative and absolute pupil dilation.

Relative pupil dilation						
10	20	30	40	50	60	Speed (degree/s)
0.010	0.046	<0.001	0.010	<0.001	<0.001	0
	1.000	0.087	0.813	0.021	0.029	10
		1.000	1.000	0.240	0.326	20
			1.000	0.334	1.000	30
				1.000	1.000	40
					1.000	50
Absolute pupil dilation						
10	20	30	40	50	60	Speed (degree/s)
0.010	0.047	<0.001	0.010	<0.001	<0.001	0
	1.000	0.081	0.783	0.021	0.027	10
		1.000	1.000	0.236	0.314	20
			1.000	0.349	1.000	30
				1.000	1.000	40
					1.000	50

### Experiment 2

To further evaluate the pupillary response difference, different stimuli were designed to target either the cone- or rod-mediated pathway. The pupil diameter and pupil dilation results across all speeds and the p values of the comparisons between speeds on the relative and the absolute pupil dilation for the cone and rod paradigms are shown in Table 3 and Table 4. The absolute pupil dilation and relative pupil dilation both demonstrated that the motion stimulus induced a significant dilated pupillary response at speeds of 10, 20, 30, 40, 50 and 60 degrees/s compared with the static pupil size in both rod- and cone-mediated paradigms (p<0.05, respectively). The motion stimulus induced pupil dilation in a speed-dependent manner in rod- and cone-mediated paradigms as the pupil dilation increased as the speed increased, and the tendency was the same for the absolute and relative pupil dilation.

**Figure 3.**
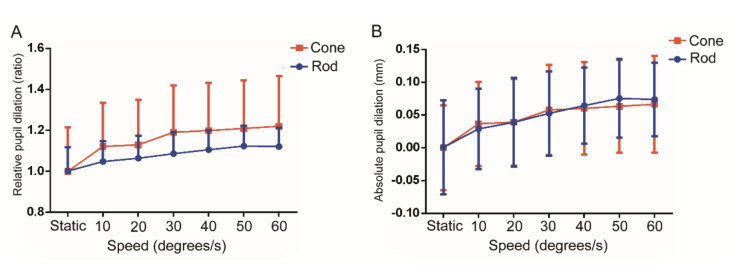
The relative pupil diameter with static optotypes and moving optotypes at the speeds of 10, 20, 30, 40, 50, and 60 degrees/s in the cone- and rod-mediated paradigms (n=28). A. The relative ratio of pupil diameters; B. Absolute value of pupil diameter change.

Further analysis showed no significant difference in the absolute pupil dilation between the cone- and rod-mediated paradigms at all speeds (p>0.05, respectively). However, the relative pupil dilation in the cone-mediated paradigm was significantly larger than that in the rod-mediated paradigm (p<0.001), and the statistical significance was shown for all speeds (p<0.05) except for 30 degrees/s (p=0.055), as shown in **Figure 3**. As shown in **Table 4**, considering the significant difference in the pupil dilation between two adjacent speeds, a post hoc analysis between speeds was performed. And the results demonstrated that the increase in the absolute and relative pupil dilation stopped at 40 degrees/s for the rod-mediated and at 30 degrees/s for the cone-mediated paradigm, which meant that the increased pupil dilation in the cone-mediated pathway reached saturation with speed slower than in the rod-mediated pathway.

**Table 3.** Pupil diameter and pupil dilation of all speeds.

Rod paradigm			
Speed	PD (mm)	APD (mm)	RPD (ratio)
(degree/s)	mean (SD)	mean (SD)	mean (SD)
Static	6.12 (0.72)	0.005 (0.72)	1.0 (0.117)
10	6.4 (0.61)	0.29 (0.61)	1.05 (0.1)
20	6.5 (0.68)	0.39 (0.68)	1.063 (0.11)
30	6.63 (0.64)	0.52 (0.64)	1.086 (0.105)
40	6.75 (0.58)	0.64 (0.58)	1.105 (0.095)
50	6.86 (0.6)	0.75 (0.6)	1.123 (0.099)
60	6.85 (0.56)	0.74 (0.56)	1.12 (0.092)
Cone paradigm			
Speed	PD (mm)	APD (mm)	RPD (ratio)
(degree/s)	mean (SD)	mean (SD)	mean (SD)
Static	3.02 (0.65)	0.00 (0.65)	1.0 (0.214)
10	3.39 (0.64)	0.37 (0.64)	1.121 (0.212)
20	3.41 (0.66)	0.39 (0.66)	1.129 (0.219)
30	3.6 (0.69)	0.58 (0.69)	1.19 (0.228)
40	3.62 (0.7)	0.6 (0.7)	1.198 (0.233)
50	3.65 (0.71)	0.63 (0.71)	1.209 (0.234)
60	3.68 (0.74)	0.66 (0.74)	1.22 (0.245)

Note: SD, standard deviation; PD, pupil diameter; APD, absolute pupil dilation; RPD, relative pupil dilation

**Table 4.** P-value of comparison between speeds for relative and absolute pupil dilation in cone and rod paradigm.

Relative pupil dilation in rod paradigm (ratio)						
10	20	30	40	50	60	Speed (degree/s)
0.003	<0.001	<0.001	<0.001	<0.001	<0.001	0
	1.000	0.003	<0.001	<0.001	<0.001	10
		0.037	<0.001	<0.001	0.003	20
			0.006	0.002	0.072	30
				1.000	1.000	40
					1.000	50
Relative pupil dilation in cone paradigm (ratio)						
10	20	30	40	50	60	Speed (degree/s)
0.003	0.003	<0.001	<0.001	<0.001	<0.001	0
	1.000	0.120	0.075	0.028	0.016	10
		.033	0.007	0.002	0.001	20
			1.000	1.000	1.000	30
				1.000	1.000	40
					1.000	50
Absolute pupil dilation in rod paradigm						
10	20	30	40	50	60	Speed (degree/s)
0.003	<0.001	<0.001	<0.001	<0.001	<0.001	0
	1.000	0.003	<0.001	<0.001	<0.001	10
		0.034	<0.001	<0.001	0.003	20
			0.006	0.002	0.073	30
				1.000	1.000	40
					1.000	50
Absolute pupil dilation in cone paradigm						
10	20	30	40	50	60	Speed (degree/s)
0.003	0.003	<0.001	<0.001	<0.001	<0.001	0
	1.000	0.124	0.082	0.029	0.017	10
		0.032	0.007	0.002	0.001	20
			1.000	1.000	1.000	30
				1.000	1.000	40
					1.000	50

## Discussion

The pupillary motion response has been indicated in previous research (4; 43). The present research investigated the pupillary response when observers were pursuing an object moving at different speeds. We demonstrated that the motion stimulus induced pupil dilation in a speed-dependent pattern. Specifically, as the speed increased, the pupil dilation gradually increased and tended to reach saturation. Further studies applied different text paradigms to target the cone- or rod-mediated pathway. The results showed that motion stimuli in the cone and rod paradigms similarly led to pupil dilation as observed in experiment 1. In addition, the increased pupil dilation in the cone paradigm became saturated with a speed slower than the rod paradigm.

Motion preparation refers to the readiness for responding before the motion of stimuli. Completing motion vision involves complicated eye tracking, including smooth pursuing movements, saccades, and others, to maintain the image on the retina adjacent to the fovea to create a clear vision (24). Thus, saccade preparation has been investigated and casts light on visual motion perception preparation (39). Previous research has identified that the superior colliculus (SC) and frontal eye field (FEF) project directly to the paramedian pontine reticular formation that induces saccades (44). Additionally, a transient pupil dilation could be induced by stimulating the rostral and caudal SC(40). Thus, the SC might connect to the pupil control network, and saccade initiation might cause a pupil response.

Previous research has demonstrated that pupil dilation occurred before a saccade during a short period in both pro-and anti-saccade tasks as motor preparation. (12; 27; 50) In the current study, following the presentation of a static stimulus in the center of the screen, participants needed a saccade to track the trajectory of moving stimuli on the left side of the screen. Due to instructions and pre-training, the observer could predict the switch between the static and moving optotypes, and preparatory processes began before the saccade onset. Thus, the pupillary response might be caused by the saccade preparatory process. In this paradigm, the disappearance of the fixation target might have served as a signal that initiated the preparation set, during which the fixation-related neurons reduced their activity and the saccade-related neurons responded, including the SC and FEF, which might link to the pupil control circuit that results in pupillary dilation.

In addition to saccade preparation, the motion-induced pupil dilation might be related to percept transitions in the current research. Well-designed studies have identified that pupil responses could be elicited by cognitive processes, including attention, decision-making, perceptual selection and so on (13; 15; 51). A previous study demonstrated the pupil dilation induced by bistable moving stimuli around the time of a percept switch, and a subsequent controlled experiment attributed the pupillary response to motor response and perceptual transition (26). The norepinephrine (NE)-releasing structure, the locus coeruleus (LC), might play a critical role here and has been demonstrated to be involved in pupil-related cognitive processes (3). Research has shown that the EW nucleus received direct input from the LC, serving as an inhibitory factor, and pupil responses might be related to LC-regulated arousal that spread through the reticular activating system (38). In the present study, following the observation of a static optotype, participants were required to start pursuing the moving target as closely as possible. During the transition from the static to dynamic optotypes, observers need percept switch and shift attention to better catch up with the moving optotypes, and the perceptual transition might be transmitted through the LC to the EW nucleus, inducing pupil dilation.

Previous research has investigated the effect of pupil size on dynamic visual acuity and demonstrated that dynamic visual acuity significantly improved following pupil dilation and decreased following pupil constriction (49). The study attributed the dilated pupil induced improvement in dynamic visual acuity to peripheral retina awareness and the application of rods. The investigation of the distribution of retina cells showed that rods and M ganglion cells were both located more peripherally (10). The present research demonstrated that pupil dilation responded to moving objects and that the dilation increased as the moving speed increased. These results suggested that pupil dilation could promote the application of the peripheral retina to elicit better dynamic vision. That is, pupil dilation is not only a result of motion perception but also a factor improving motion perception, which forms a feedback loop.

The pupil response results from a complicated interrelationship between the sympathetic and parasympathetic pathways that receive multiple inputs from the cortical and subcortical areas. As a reflection of the cortical processes to sensory and nonsensory stimuli, pupil size responds in a stimuli-dependent pattern (9). Previous research has demonstrated that the anti-saccade task-induced greater pupil dilation than the pro-saccade task (50), and saccade initiation-associated area FEF and SC activation was higher in the anti-saccade task, as shown by fMRI (8) and single neuron recording(16). Additionally, a previous study showed that saccade preparatory activity was negatively associated with saccade reaction time (2; 14), and pupil diameter changes were negatively correlated with saccade latency or with saccade reaction time (36). These results indicated that pupil dilation is associated with cortical processing related to saccade preparation.

The present study demonstrated that the motion stimuli led to pupil dilation in a speed-dependent pattern, which indicated that pupil dilation increased as the speed increased. Electrophysiology studies have shown that the stimuli with increasing speed result in shorter latencies and larger amplitudes in the visual pathway recorded by visual evoked potential (25). The results suggested that higher speed stimuli might induce quicker and more active visual motion perception input that might better activate the saccade preparation-related neurons and shorten saccade latency. Thus, pupil dilation increased as speed increased. However, there is an inflection point regarding human speed identification capacity (47). In addition, electrophysiological studies have revealed that if the speed continues to increase, the optimal speed appears where the N2 amplitude reaches its peak, and the potential will not increase at the greater speed (25). Thus, the latency will not be shorter after this inflection point, and pupil dilation becomes saturated. And the size of the dilated pupil and the activity of pupil-regulating muscle fibril are limited. Thus, the present research showed that as the speed increased, the tendency for the increase in pupil dilation gradually slowed down and became saturated.

As the starting point of visual motion signal input, the retina comprises three layers, including the cones and rods, bipolar cells and ganglion cells. As mentioned in the Introduction, rods primarily project into the magnocellular LGN layers, while its input to the P-pathway cells is weak. (32; 33; 41) Given the critical role of the M ganglion cell pathway in motion vision and the specified projection relationship between rods and the M pathway, rods may play a crucial role in visual motion input. Thus, we hypothesized that motion stimuli targeting cone- or rod-mediated pathways might induce different pupillary response patterns. In the current research, the cone and rod paradigms were capable of inducing pupil dilation in a speed-dependent pattern similar to experiment 1. The results indicated that the visual motion perception input could be individually transmitted through either the cone- or rode-mediated pathways. A previous study showed that rods and cones project to the same ganglion cells (32; 33; 41), and the signal was integrated into ganglion cells that subsequently transmitted the signal to cortical areas involved in motion perception.

Additionally, the current research demonstrated that the relative pupil dilation, but not the absolute pupil dilation, in the cone paradigm was larger than in the rod paradigm. The pupil light reflex is the fundamental function of the pupil in regulating light influx. In normal circumstances, the pupil size in the photopic environment was significantly smaller than that in the scotopic environment. Thus, the larger absolute value of pupil dilation for the cone paradigm might be partially attributed to the smaller baseline pupil size. Post hoc analysis across different speeds in the present study demonstrated that pupil dilation in the rod paradigm increased as the speed increased and reached saturation at 40 degrees/s, and the speed was faster than that in the cone paradigm, which was 30 degrees/s. The results indicated that the rod-mediated pathway seems less sensitive to the visual motion perception input, despite rods projecting mainly to the M pathway. Accordingly, previous research on dynamic vision under scotopic and photopic conditions demonstrated that the fusion frequency of rod-mediated vision was significantly lower than that of cone-mediated vision (47). The research has indicated that motion identification and detection sensitivity decreased with reduced luminance (53). The disparity in motion perception in different luminance conditions might be attributed to the distinct motion sensitivity of the cone- and rod-mediated pathways. Research had demonstrated that when the same stimuli activated rods, they appeared to move 20-25% slower than when activating cones (20); this might provide a basis for the increased pupil dilation inflection point in the rod paradigm, which was approximately 10 degree/s slower than in the cone paradigm because there was approximately 20% disparity in the speed perception. In addition, the cones are mainly distributed in the fovea, and the rods are more peripherally located. It can be speculated that as the pupil enlarged to a certain extent in the cone paradigm, the marginal benefit of pupil dilation would decrease as the amount of additionally activated cones decreased. However, the enlarged pupil size could still involve more rods due to their distribution. Thus, pupil dilation continued to increase in the rod paradigm after the saturation speed was reached in the cone paradigm.

Certain limitations exist in the present study. Due to the limitation in devices, image capturing was not continuous and the calculated pupil size did not consider gazing and elliptical fits, which was less accurate than the pupillometer with eye-tracking function. Additionally, an electrophysiological examination was not performed in the present research, which led to a lack of a theoretical basis for explanation. Moreover, the motion speeds of the stimuli were present in a fixed increasing sequence rather than randomized, which might induce a sequential effect on pupil dilation. The change in pupil dilatation across target speeds might reflect additional confounding factors rather than speed manipulation. We only observed horizontal motion with a specific spatial frequency, and the effect of pupillary changes may be different in other motion patterns. Further research on motion perception-related pupillary responses should pay more attention to the neuronal networks in the brain and disparities in the responses based on the differences in the stimuli.

To conclude, motion stimuli induced pupil dilation in a speed-dependent manner, and as the motion speed increased, the pupil dilation gradually increased and became saturated. In addition, the absolute value of pupil dilation but not the dilation ratio caused by the motion stimuli targeting the cone-mediated pathway was larger compared with the rod-mediated pathway. Pupil dilation induced in the cone paradigm became saturated more quickly than that in the rod paradigm.

### Ethics and Conflict of Interest

The author(s) declare(s) that the contents of the article are in agreement with the ethics described in http://biblio.unibe.ch/portale/elibrary/BOP/jemr/ethics.html and that there is no conflict of interest regarding the publication of this paper.

### Acknowledgements

This work was supported by the grant from Chinese Capital’s Funds for Health Improvement and Research (Grant number: CFH2018-2-4093).
